# Improving Quality of Care via Effective Pain Management

**DOI:** 10.1093/geroni/igab046.3225

**Published:** 2021-12-17

**Authors:** Jennifer Rose

**Affiliations:** The College of St. Scholastica, Foxboro, Wisconsin, United States

## Abstract

Pain is neither a vital sign nor a normal part of aging. Yet, older adults frequently experience pain chronically or from an acute event. Pain was identified as a gap per the Centers for Medicare & Medicaid Services Quality Measures report (2019). The purpose of this quality improvement project was to improve the assessment of pain at a skilled nursing facility (SNF) by using a standardized tool. The Comprehensive Pain Assessment Tool for the Cognitively Intact evaluates the complex sensation and emotional reaction of the pain experience. Nurse managers (N=7) received 1:1 education on pain, pain assessment, use of the pain assessment tool, and took a post-test. Chart audits were conducted to identify tool use and evaluate the patient response. Additional data were collected from nurse managers via a questionnaire. All nurse managers received education and completed the post-test. Pain assessments and care plans were completed for 100% of the SNF residents in the cohort (N=22). Follow-up assessments were completed on only 75% of the cohort. Of the cohort, 95% demonstrated improved physical ability and functioning in activities of daily living as their pain experience improved. Only 4.5% of the cohort participated in the anticipated level of minutes of therapy as a result of facility infection control limitations due to the COVID-19 pandemic. This project demonstrated improved pain management through use of a tool to comprehensively assess pain. An organizational policy to comprehensively assess pain at this SNF could promote a higher level of independence and functioning for older adults.

